# Memory-focused therapy: an integrated intervention to reduce trauma symptoms, maladaptive cognitive processes, and emotional distress in Afghan youth

**DOI:** 10.3389/fpsyt.2026.1825376

**Published:** 2026-07-08

**Authors:** Sayed Jafar Ahmadi, Zeinab Musavi, Sayed Abbas Sarwary, Delawar Khan Ebrahimi, Adam D. Brown, Justin Dainer-Best, Laura Jobson

**Affiliations:** 1 Psychology Program, Bard College, Annandale-on-Hudson, NY, United States; 2 Turner Institute for Brain and Mental Health & School of Psychological Sciences, Monash University, Melbourne, Australia; 3 Department of Psychology, The New School, New York, NY, United States; 4 Student Affairs Office, American University of Afghanistan, Doha, Qatar; 5 Deportment of Research, Behrawan Research and Psychology Services Organization, Kabul, Afghanistan; 6 Department of Medical Sciences, Kateb University, Kabul, Afghanistan; 7 Department of Psychiatry, New York University (NYU) Grossman School of Medicine, New York, NY, United States

**Keywords:** Afghan youth, cognitive processes, memory-focused therapy, posttraumatic growth, PTSD, resilience, war trauma

## Abstract

**Background:**

Afghan youth continue to face chronic war-related trauma, terrorist violence, and severe disruptions to education and social support systems, resulting in high rates of posttraumatic stress disorder (PTSD), depression, anxiety, and hopelessness. There is a critical need for culturally responsive, low-intensity, and feasible psychological interventions that can be delivered in low-resource and unstable settings.

**Objective:**

This study conducted a preliminary evaluation of the efficacy, acceptability, and mechanisms of change associated with Memory-Focused Therapy (MFT), an integrative intervention targeting autobiographical memory processing, acceptance-based regulation, and future self-construction among youth affected by the Kaaj Education Center attack in Kabul, Afghanistan.

**Methods:**

A single-group repeated-measures design was used with 26 participants assessed at baseline, post-intervention, and three-month follow-up. Standardized measures of PTSD, depression, anxiety, stress, cognitive avoidance, cognitive fusion, resilience, and posttraumatic growth were administered. MFT was delivered in 12 structured group sessions. Additionally, qualitative data from semi-structured interviews and therapist field notes were analyzed using thematic analysis.

**Results:**

Quantitative analyses showed significant reductions in PTSD symptoms, depression, anxiety, stress, cognitive avoidance, and cognitive fusion from baseline to post-intervention, alongside significant increases in posttraumatic growth. Several of these improvements were maintained at follow-up. Qualitative findings reflected four overarching themes: (1) facilitator experiences and implementation challenges in high-risk contexts, (2) improvements in cognitive and emotional processing, (3) growth in meaning, relationships, values, and future orientation, and (4) exposure to traumatic memories and reduced avoidance.

**Conclusion:**

Findings provide preliminary evidence that MFT is a feasible, acceptable, and potentially effective intervention for trauma-affected Afghan youth. By integrating trauma memory processing, present-moment emotional regulation, and future-oriented meaning-making, MFT appears to support improvements in psychological coherence, self-continuity, and resilience. Further controlled and longitudinal studies are needed to confirm these effects and examine underlying mechanisms.

Afghanistan continues to contend with one of the world’s worst humanitarian crises (1, 2). Afghan people have endured decades of war, conflict, natural disasters, social injustices, human rights violations, and poverty (3, 4). These circumstances have resulted in significant psychological suffering, resulting in a mental health crisis (4–6). Many Afghans, including youth, are suffering serious mental health concerns, including elevated levels of posttraumatic stress disorder (PTSD), depression and anxiety, which interfere with daily functioning (3, 4, 7). Despite this concerning need, “Afghanistan’s mental health infrastructure is critically underdeveloped” (4, p.2), with a severe shortage of health professionals (only around 100 psychiatrists nationwide and 0.35 psychologists per 100,000 people) and a treatment gap over 90% (i.e., over 90% of individuals who require mental health care do not receive adequate psychological or psychiatric treatment, reflecting severe shortages in mental health infrastructure and workforce) (4, 8). Compounding this, since the Taliban regained control in 2021, mental health access has further deteriorated (7–9). Therefore, there is an urgent need for evidence-based psychological approaches, including for youth, that can be implemented in this region to promote health equity and sustainable systems of health care (5).

Here we introduce a novel, evidence-based therapeutic approach for youth in conflict-affected and low-resource settings, Memory-Focused Therapy (MFT). MFT was developed by the lead author, following two decades of clinical research in Afghanistan and with Afghan refugees in Pakistan and Iran. This intervention aims to target cognitive disruptions known to trans-diagnostically underpin PTSD and depression (10), specifically, the processing of personal autobiographical memories, emotional processing, acceptance of the present, and prospective memory of the future. The primary goal of MFT was not only to reduce symptoms of PTSD and depression, but also to help individuals reconstruct the self, strengthen resilience and promote post-traumatic growth. The primary targets of MFT are presented in **Table 1** and discussed below.

**Table 1:** Primary targets of memory focused therapy.

Primary targets of MFT	Rationale
Autobiographical memory disruptions and future thinking	Addresses the cognitive–emotional processes that shape identity, continuity of self, and resilience across time.
Memory specificity	Functions as a cognitive marker and predictor of the course and severity of depression and PTSD.
Acceptance and mindfulness	Enhances psychological flexibility and promotes acceptance of internal experiences, reducing avoidance and cognitive fusion.
Prospective memory, episodic future thinking, and meaning-making	Supports future planning, fosters hope, and creates opportunities for reflection on personal meaning, goals, and values.

First, MFT targets memory disruptions, which are critical to treating PTSD and depression. Autobiographical memory and future thinking form a cognitive–emotional cycle that shapes not only identity but also vulnerability and resilience (10, 11). Emotions influence the encoding, organization and recall of autobiographical memory (12). Intense emotion, particularly related to trauma, has the potential to disrupt the autobiographical memory structure and impair the verbally accessible and the sensory–somatic memory systems (e.g., 13, 14). Therefore, disruptions in the trauma memory are a hallmark symptom of PTSD (15). Consequently, PTSD theories focus on memory (15–18) and evidence-based psychological interventions target the processing of the trauma memory (19). Moreover, at a transdiagnostic level, memory disruptions also contribute to other psychological disorders, such as depression, anxiety, obsessive-compulsive disorder (OCD), and suicidal ideation (e.g., 20). Therefore, MFT included a focus on the processing of trauma memories.

Second, in both PTSD and depression individuals often have difficulties recalling specific memories (i.e., detailed memories of events that occurred at specific time and place) and instead recall over general memories (OGMs; 21). OGM emerges from a combination of rumination, emotional avoidance, and impaired executive functioning, maintaining a cycle of general recollections, negative affect, and helplessness (21). OGM serves to avoid painful emotions linked to specific events and is a cognitive marker and predictor of the course of depression and PTSD (22–25). OGM is also commonly observed in youth (26), including those exposed to war-related trauma (27). Reduced memory specificity has important cognitive consequences, including impaired problem-solving, difficulty imagining specific future events, rumination, reduced exposure to negative memories necessary for long-term well-being, and difficulty accessing trauma-related information essential for PTSD recovery (21, 23, 27). These deficits can contribute to ongoing negative social interactions, low self-efficacy, hopelessness, and symptom maintenance (21, 23, 27, 28). Importantly, Memory Specificity Training (MEST), which aims to increase specific autobiographical recall, has been found to improve memory specificity and reduce symptoms of PTSD and depression (23, 27, 29, 30). Recent reviews highlight MEST’s promise for treating psychopathology and its feasibility in mental health services (31). Therefore, MFT also included a focus on memory specificity.

Third, MFT targets acceptance and mindfulness. Acceptance and Commitment Therapy (ACT) improves mental health by enhancing psychological flexibility and acceptance of internal experiences (32, 33). A recent randomized controlled trial with Afghan adolescent girls found that while both ACT and trauma-focused cognitive behavioral therapy (TF-CBT) were effective in treating PTSD, ACT produced a larger effect size for reducing intrusions (*d* ≈ 3.07 vs. *d* ≈ 2.55), with 70% of participants falling below the PTSD cutoff following ACT, compared to 55% in the TF-CBT group (34). Additionally, ACT was found to be more culturally acceptable. Therefore, MFT also included important components of ACT, including psychological flexibility, mindfulness, and strategies to enhance acceptance.

Fourth, MFT targets planning for the future and hope. Prospective memory can reflect the quality of autobiographical memory; individuals with poor memory specificity are less capable of imagining positive future events, and this deficit can lead to hopelessness and disruptions in constructing a “future self”, which in turn manifest as PTSD and depression symptomatology (35, 36). Moreover, the “fading affect bias”—which naturally weakens negative emotional memories over time—is diminished or reversed in depression and PTSD, causing negative memories to remain salient and positive memories to fade (37). This has significant consequences for youth being able to imagine a future. Therefore, MFT included a focus on episodic future thinking, values and meaning-making.

MFT is based on two decades of research demonstrating that memory-based interventions, through targeting processing of the trauma memory, memory specificity, episodic future thinking, and bodily memory, can significantly reduce symptoms of PTSD, depression, anxiety, and complicated grief (27, 29, 30). In the trauma field, Narrative Exposure Therapy (NET), Written Exposure Therapy (WET), and Writing for Recovery reorganize trauma memories by transferring content from the sensory–somatic system to the verbal-linguistic system, enhancing temporal and emotional coherence and reducing PTSD symptoms among refugees and war survivors (3, 38–40). Imagery-based interventions, such as Imagery Rescripting and Episodic Future Thinking Training (FT-Training), reduce core negative beliefs, hopelessness, and passivity, while strengthening episodic future thinking, goal-directedness, and a healthier future self (22, 36, 41, 42). Body-based approaches, such as Eye Movement Desensitization and Reprocessing (EMDR) and somatically oriented interventions, reduce threat-system hyperactivation and integrate bodily memory with narrative processing that can improve memory structure and emotional regulation (43). However, many of these evidence-based approaches face limitations in low-resource settings, such as Afghanistan (44).

MFT was developed to incorporate these key aspects known to underpin clinical change in a context like Afghanistan, where there are difficulties accessing services, few specialist providers, limited resources and intense trauma exposure can lead to treatment resistance or dropout (34). Since 2010, our team has focused on developing and evaluating brief, low-intensity trauma interventions among war-affected Afghan adolescents and youth, including WET (3), MEST (45, 46), psychological debriefing (46), TF-CBT (3, 34, 45, 46), ACT (34), Memory Training for Recovery- Adolescent (METRA) (47, 48), and METRA+ (49). With the exception of psychological debriefing, these interventions have been effective in reducing symptoms of PTSD and depression. However, clinical experience and qualitative feedback indicated that several of these approaches—particularly psychological debriefing, TF-CBT, and WET—were experienced as difficult, rigid, and culturally distant from the lived experience of many Afghans youth. In Afghan culture, direct emotional expression and sustained focus on the “self” conflicts with norms of modesty, fear of judgment, and collectivistic values.

Our work with ACT has highlighted strong cultural resonance, with adolescents describing it as “more joyful,” “closer to our culture,” and “less threatening” (34). Concepts, such as fate, destiny, and meaning-making in suffering, which are highly salient in Afghan culture, when channeled within ACT toward active acceptance, values, and meaningful action, reduced excessive self-blame, while strengthening a sense of agency (34). In recent years, with ongoing trauma, hopelessness about the future, disruption of “self-continuity,” and increases in suicidal thoughts and behaviors among Afghan youth, the idea of strengthening self-continuity and designing a therapeutic approach that could work simultaneously on the past (e.g., processing of memories), present (e.g., acceptance, emotion regulation, distress tolerance), and future (e.g., goal-setting, constructing a future self) became necessary. Accordingly, the primary aim of MFT was not only to reduce PTSD and depression, but to restore self-continuity and foster durable resilience by reconnecting memory processing, present-moment regulation, and future self-construction, positioning MFT as an evidence-based, culturally attuned, and feasible intervention in low-resource Afghan contexts.

## Current study

The aim of this study was to conduct a preliminary evaluation of the efficacy and acceptability of MFT among youth affected by the explosion at the Kaaj Education Center in Kabul. In late 2022, with far-reaching consequences into early 2023, the terrorist attack on the Kaaj Education Center in West Kabul led to the deaths of at least 53 students—mostly girls—and injured more than 110 others (50–52). This study sought to determine whether implementing MFT was associated with significant reductions in PTSD and depressive symptoms and increases in resilience and post-traumatic growth compared to baseline. We also aimed to examine whether MFT was deemed acceptable for Afghan youth. Based on previous evidence regarding memory-based interventions, it was hypothesized that MFT, by integrating a focus on the past–present–future, would reduce symptoms and help reconstruct self-continuity and strengthen resilience (27, 47, 48, 53).

## Methods

### Study design

This study employed a single-group repeated-measures approach. The study protocol was approved by the Ethics Committee of Kateb University (Approval No: MRC-KU-2022-00112). Informed written consent was obtained from all participants prior to participating in the study. Assessments were conducted at three timepoints: (1) baseline, (2) post-intervention, and (3) three-month follow-up. The aim of this design was to examine changes in PTSD symptoms, depression, anxiety, stress, resilience, and post-traumatic growth.

### Participants

Participants were youth aged 18 to 22 years (*M* = 19.19, *SD* = 1.27) who were direct survivors of the explosion at the Kaaj Education Center. Inclusion criteria were: (a) scoring above the clinical cutoff for PTSD on the Impact of Event Scale-Revised (IES-R ≥ 33; 54), (b) confirmed PTSD diagnosis based on a structured clinical interview, (c) willingness to participate in 12 treatment sessions, (d) no current psychotropic medication use, psychotic disorders, or severe cognitive impairment, and e) not receiving concurrent psychotherapy or pharmacotherapy during the study period. A total of 82 individuals were referred for psychological support by the administration of the Kaaj Education Center. Following initial screening with the IES-R, 42 individuals scored above the clinical cutoff. Of these, 26 met the full eligibility criteria and these individuals were enrolled in the study.

### Procedure

Following an introductory session in which the study aims were explained, written informed consent was obtained. Baseline assessments were then completed. The MFT intervention consisted of 12 group-based sessions, each lasting 75–90 minutes, with groups of 8–10 participants. Post-intervention assessments were completed immediately after the final MFT session, and follow-up assessments were conducted approximately three months later. All assessments were conducted by an assessor blind to the study aims. Confidentiality was ensured throughout the study, and participants were informed that they could withdraw at any time without penalty. All ethical requirements and institutional guidelines were adhered to. After completing the intervention, participants’ perspectives and experiences were explored through semi-structured interviews. In addition, facilitators’ reports and observational notes were collected and analyzed. To further examine potential longer-term effects, supplementary follow-up interviews were conducted with five participants two years after the intervention. Qualitative data were analyzed using thematic analysis with MAXQDA 24 software.

### Measures

#### Clinical symptoms: PTSD, depression, anxiety and stress

##### Impact of event scale–revised

The IES-R (55) is a 22-item self-report measure assessing PTSD symptoms. Items were rated on Likert scales ranging from 0–4. A total score of 33 is widely recommended as the clinical cutoff for probable PTSD (Creamer et al., 2003) (54). The IES-R demonstrates strong construct and convergent validity across cultures, including conflict-affected and Afghan populations (56) and has excellent internal consistency (α = .88–.94) with test–retest reliability ranging from.79 to.87 (55). In this study internal consistency was good (Cronbach’s alpha=.83).

##### Depression anxiety and stress scale

The DASS-42 (57) is a 42-item self-report measure assessing depression, anxiety, and stress across three 14-item subscales. Items were rated on 0–3 Likert scales. The measure has strong factorial validity and excellent internal consistency (α = .89–.95) and is widely used in trauma-exposed populations including among Afghans (6). In this study internal consistency was good (Cronbach’s alpha=.87).

#### Cognitive processes: cognitive avoidance and fusion

##### Cognitive avoidance questionnaire

The CAQ is a self-report measure originally developed in French as the Questionnaire d’Évitement Cognitif (QEC; 58) and consists of 25 items assessing five cognitive avoidance strategies: thought suppression, thought substitution, distraction, avoidance of threatening stimuli, and transformation of images into thoughts. The English version validated by Sexton and Dugas (59) demonstrated a robust five-factor structure, strong internal consistency (α = .85), good temporal stability and solid convergent and divergent validity. In this study internal consistency was good (Cronbach’s alpha=.74).

##### Cognitive fusion questionnaire

The CFQ (60) is a self-report measure of cognitive fusion—rigid entanglement with one’s thoughts—which is a central construct within ACT. The seven items were rated on 1–7 Likert scales (“*never true*” to “*always true*”). Internal consistency has been found to be excellent (α = .86–.92), with strong convergent validity with measures of depression, anxiety, and experiential avoidance (60) and has been found to have strong psychometric properties among Persian samples (e.g., 61). In this study internal consistency was good (Cronbach’s alpha=.82).

#### Growth outcomes: emotional resilience and posttraumatic growth

##### Ego resilience scale

The ERS (62) assessed emotional–cognitive flexibility and the capacity to recover from stress. The 14 self-report items were rated on 1–4 Likert scales (“*not true at all*” to “*very true*”). Internal consistency typically ranges from.75 to.85, and the scale shows strong convergent validity with psychological well-being, coping, and social adaptability (62). In this study internal consistency was good (Cronbach’s alpha=.81).

##### Posttraumatic growth inventory

The PTGI (63) is a self-report measure that assessed positive psychological changes following trauma across five domains, including personal strength, relating to others, spirituality, appreciation of life, and new possibilities. The 21 items were rated on 6-point rating scales, with higher scores indicating greater growth. The PTGI demonstrates strong construct validity and high internal consistency (α = .89–.92) (63) and has been used in Persian samples (64). In this study internal consistency was good (Cronbach’s alpha=.86).

### Intervention

The 12-session MFT intervention was structured along a sequential pathway. Session 1 focused on life-mapping and group introduction. Session 2 provided psychoeducation on trauma, recognition of PTSD symptoms and mind–body connections. In Session 3, participants were introduced to the concepts of avoidance and graded exposure and taught the “butterfly hug” technique. Session 4 focused on different types of memory, autobiographical memory, and specific-memory recall exercises. Session 5 included narrative reconstruction, encompassing self-compassion, mindfulness practice, and emotion labeling. Sessions 6 and 7 addressed cognitive diffusion, acceptance, acceptance-based metaphors, and letting go of excessive control. In Session 8, the emphasis shifted to values clarification and writing a ten-year future vision. Session 9 highlighted meaning-making, post-traumatic growth and gratitude. Session 10 involved SMART goal-setting, future planning, and communication skills. Session 11 centered on integrating skills, rewriting one’s life story, and strengthening future-oriented resilience. Session 12 concluded the program with a review of achievements, discussion of potential future challenges, and a formal closing session.

### Data analysis plan

Data were analyzed using SPSS (Version 27). To examine the effectiveness of the MFT intervention over time, a series of linear mixed-effects models were conducted separately for each outcome variable, including PTSD symptoms, depression, anxiety, stress, cognitive avoidance, cognitive fusion, emotional resilience, and posttraumatic growth. Time was entered as a fixed within-subject factor with three measurement points (baseline, post-intervention, and three-month follow-up). Participant was included as a random intercept to account for within-subject dependency across repeated assessments. An unstructured covariance matrix was specified to model the correlations among repeated observations across time. Model parameters were estimated using Restricted Maximum Likelihood (REML). Degrees of freedom were estimated using the Satterthwaite approximation. Missing data were handled under the Missing at Random (MAR) assumption, which is accommodated within the mixed-effects modeling framework. Pairwise comparisons of estimated marginal means were performed using the Least Significant Difference (LSD) method. Statistical significance was set at *p* <.05. Effects are reported as mean differences (MD), standard errors (SE), and 95% confidence intervals (CI).

### Qualitative data analysis

Qualitative data consisted of a) semi-structured interviews conducted after the completion of the intervention with all participants, b) facilitators’ observational notes collected across the 12 sessions in 2022, and c) follow-up interviews with five participants two years post-intervention. All qualitative materials were audio-recorded and transcribed verbatim. The qualitative data was analyzed using reflective thematic analysis (Braun & Clarke, 2019) (65), supported by MAXQDA 24 software.

The analytic process followed a structured, multi-stage procedure to enhance rigor and reliability (65). First, the research team engaged in repeated reading of transcripts and field notes to achieve familiarity with the data and to develop an initial holistic understanding of participants’ experiences. Second, two researchers independently conducted initial coding by identifying meaningful units, phrases, and expressions that captured core elements of participants’ narratives. Third, through an iterative process, related initial codes were organized into more abstract axial categories that reflected broader conceptual patterns within the data. Finally, axial categories were reviewed, refined, and synthesized into overarching themes representing central experiential domains and perceived changes following the intervention. Throughout the analysis, an audit trail was maintained to document coding decisions, theme development, and the interpretive steps taken by the research team.

## Results

### Participant characteristics

The initial sample consisted of 26 youth (19 females; 7 males). 42.1% of female participants and 71.4% of male participants reported having experienced a physical injury during the terrorist attack. Attrition occurred over the course of the study due to relocation, changes in personal circumstances, and loss to follow-up. As a result, 22 participants completed the post-test assessment, and 13 participants completed the three-month follow-up assessment (**Table 2**).

**Table 2:** Means and standard deviations of outcome measures at baseline (pre-test), post-test, and follow-up.

Outcome variable	Baseline (pre-test) *M* (*SD*) *N* = 26	Post-test M (SD) *N* = 22	Follow-up M (SD) *N* = 13
Clinical Symptoms			
PTSD	60.77 (12.19)	43.73 (20.78)	35.46 (20.95)
Depression	31.65 (6.68)	17.14 (12.64)	19.92 (13.50)
Anxiety	26.35 (8.73)	13.82 (10.67)	14.85 (13.33)
Stress	33.62 (5.15)	19.95 (12.03)	21.54 (11.63)
Cognitive Processes			
Cognitive Fusion	63.77 (17.98)	49.23 (17.86)	45.08 (19.52)
Cognitive Avoidance	96.96 (13.12)	75.05 (27.06)	72.62 (25.86)
Growth Outcomes			
Post-Traumatic Growth	50.50 (18.27)	67.32 (8.82)	57.62 (13.30)
Resilience	40.08 (8.52)	43.55 (7.31)	39.00 (8.41)

PTSD, posttraumatic stress disorder.

### Clinical symptoms: PTSD, depression, anxiety, and stress

**Figure 1:**
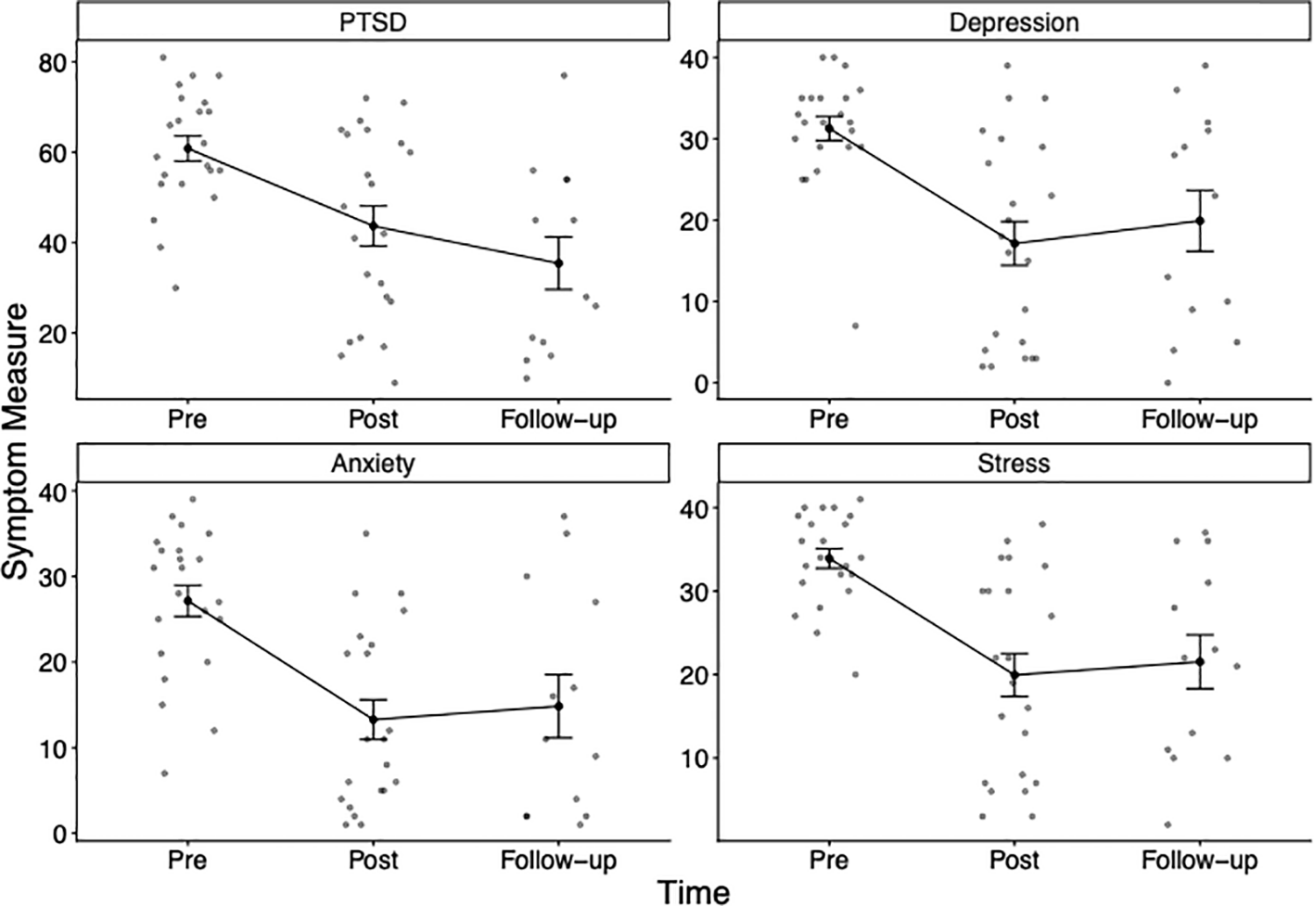
Changes in clinical symptoms before and after treatment. Symptoms improved from baseline to post-treatment and were generally stable at follow-up. PTSD symptoms were measured according to the IES-R, and symptoms of depression, anxiety, and stress were measured using the DASS-42. At baseline *n* = 26, and post-intervention timepoints, *n* = 22; for follow-up, *n* = 13.

PTSD symptoms showed a significant reduction over time, with a significant decrease from Baseline to Post-intervention (mean difference [*MD*] = -17.27, *SE* = 3.99, *p* <.001, 95% CI [-25.52, -9.03]) and from Baseline to Follow-up (*MD* = -22.88, *SE* = 4.74, *p* <.001, 95% CI [-33.14, -12.63]). (See **Figure 1** for clinical symptom change.) Depression symptoms also significantly decreased, with a significant reduction from Baseline to Post-intervention (*MD* = -14.17, *SE* = 2.36, *p* <.001, 95% CI [-19.08, -9.25]) and from Baseline to Follow-up (*MD* = -10.19, SE = 2.32, *p* = .001, 95% CI [-15.25, -5.12]). Anxiety exhibited a significant decline across time, with reductions from Baseline to Post-intervention (*MD* = -12.95, *SE* = 1.96, *p* <.001, 95% CI [-16.99, -8.91]) and from Baseline to Follow-up (*MD* = -12.01, *SE* = 2.25, *p* <.001, 95% CI [-16.62, -7.41]). Finally, stress levels decreased, with significant reductions from Baseline to Post-intervention (*MD* = -13.87, *SE* = 2.51, *p* <.001, 95% CI [-19.09, -8.65]) and from Baseline to Follow-up (*MD* = -11.89, *SE* = 2.58, *p* = .001, 95% CI [-17.53, -6.25]). For each symptom type, the difference between Post-intervention and Follow-up was not significant, suggesting symptom improvements were maintained over time.

### Cognitive processes: cognitive avoidance and cognitive fusion

Cognitive avoidance demonstrated a significant reduction from Baseline to Post-intervention (MD = -21.78, SE = 5.09, p <.001, 95% CI [-32.36, -11.20]). The difference between Baseline and Follow-up did not reach statistical significance, likely due to reduced statistical power at follow-up (*n* = 13); however, mean scores continued to decrease at follow-up (Follow-up: *M* = 72.62), suggesting maintenance of gains. There was no significant difference between Post-intervention and Follow-up. Cognitive fusion significantly decreased from Baseline to Post-Intervention (*MD* = -12.68, *SE* = 3.23, *p* = .001, 95% CI [-19.36, -5.99]) and from Baseline to Follow-up (*MD* = -15.89, *SE* = 3.90, *p* = .001, 95% CI [-24.27, -7.51]). The difference between Post-intervention and Follow-up was not significant.

### Growth outcomes: emotional resilience and posttraumatic growth

Emotional resilience showed a modest but statistically significant improvement between Baseline and Post-intervention (MD = 3.65, SE = 1.78, p = .048, 95% CI [0.03, 7.26]). However, no other comparisons reached statistical significance, suggesting that these findings should be interpreted with caution. In contrast, posttraumatic growth demonstrated stronger and more sustained increases, with significant improvements from Baseline to Post-intervention (MD = 16.85, SE = 3.81, p <.001, 95% CI [9.08, 24.62]) and from Post-intervention to Follow-up (MD = 9.81, SE = 3.68, p = .019, 95% CI [1.90, 17.72]).

### Qualitative findings

In total, 241 meaning units were analyzed. This analytic process resulted in the identification of four overarching themes, each representing distinct dimensions of participants lived experiences during and after the intervention. The extracted themes were as follows: 1) facilitator experiences and implementation challenges, 2) cognitive and emotional processing, 3) growth, meaning, relationships, values, and future orientation, and 4) exposure to traumatic memories and reduction of avoidance.

### Theme 1: facilitator experiences and implementation challenges

Data indicated the facilitators were actively engaged in the therapeutic process and reported a profound emotional and professional experience. One facilitator described the intervention as a dynamic and shared process; “In every session, I truly felt that I was accompanying the participants on this journey, moving together through each station of pain, suffering, and grief.” (Facilitator reflective notes). While observing gradual changes, improvements in mood and increased hope among participants constituted a central aspect of the facilitators experience, facilitators also noted that confronting participants’ initial levels of distress evoked feelings of sadness and concern.

Facilitators noted that delivering MFT in unsafe and highly stressful environments was associated with significant contextual disruptions; “The exercises in this section were disrupted due to the presence of the Taliban inside the office, which created intense fear among the girls.” (Facilitator reflective notes). Physical limitations, such as injury-related pain, physical fatigue and long travel distances to attend sessions, affected some participants’ engagement. One participant stated: “The distance to the sessions is very long, and because of my leg injury, I cannot use public transportation. As a result, the cost of transportation is very heavy for me.” (Participant 1, male).

At the session level, participants reported psychological and physical exhaustion during early sessions, particularly during repeated exposure, metaphor-based exercises, and imagery practices. Physical reactions, such as headaches, neck pain, shortness of breath, increased heart rate and difficulty closing the eyes during relaxation exercises, were frequently reported. As one participant described: “When I try to visualize my hands and feet or do relaxation exercises, I feel numbness in my left hand, and I cannot create a safe place. The safe place becomes dark for me.” (Participant 2, female). In some cases, visualizing the traumatic event elicited intense emotional reactions, including crying, distress, feelings of being “trapped under a vehicle,” or sudden intrusion of explosion-related images. Despite these challenges, participants described these difficulties as being a beneficial part of their overall experience.

### Theme 2: cognitive and emotional processing

One of the most salient themes involved meaningful changes in cognitive processing, mindfulness, mental flexibility and emotion regulation. Participants reported learning to recognize thoughts as transient mental events and gradually developing a healthier, more conscious distance from negative, anxiety-provoking, or self-critical thoughts. This shift in their relationship with thoughts—particularly in triggering situations—was described as a core therapeutic experience. One participant highlighted the practical application of this skill in an academic context:

“Before, when my English teacher asked me to read, I thought I would freeze or that other students would laugh at me. When these thoughts came, I avoided going to class. Now, even when the thoughts are there, I take a deep breath and tell myself that a thought is just a thought, and then I continue explaining the lesson. These exercises have been very helpful for me.” (Participant 3, female)

Improvements in attention, memory, and concentration were also frequently reported. As one participant stated:

“Now I can remember the details of the incident. Before, I couldn’t recall them clearly and my heart rate would increase. The incident had a negative effect on my studies and my memory was weak—I would forget where I put things. Now I realize that I had anxiety, and my memory has improved.” (Participant 2, female)

Reductions in physical symptoms, such as physiological arousal, heart palpitations, pain, dizziness and sleep disturbances, and increased bodily calm and improved emotional regulation were also reported. Participants specifically reported the benefits following deep breathing, relaxation exercises, safe-place imagery, and the butterfly hug; “After deep breathing, I feel very calm.” (Participant 4, female) and “I felt myself in the scene of the incident, but when I tapped my hands on myself (the butterfly hug), I felt calm.” (participant 13, female). Some participants noted that recalling the traumatic event no longer triggered intense bodily reactions; “When I remember the incident now, I don’t get heart palpitations like before.” (Participant 6, female) and that “Writing reduces my pain; I think my pain has decreased.” (Participant 17, female). The relaxation exercises contributed to improved sleep quality; “When I did relaxation and deep breathing exercises, I would fall asleep and feel very calm.” (Participant 4, female) and “Before, I couldn’t sleep properly, but now my sleep has improved and I fall asleep easily.” (Participant 18, female).

### Theme 3: growth, meaning, relationships, values, and future orientation

Participants reflected on their meaningful existential and value-based transformations. Participants described shifts in their worldview, reinterpretation of the traumatic event, clearer identification of personal values, and the emergence of hope for the future. In many narratives, the traumatic experience was gradually reappraised from being solely negative and debilitating to something more understandable and, in some cases, growth-promoting. One participant described:

“Before, I only thought about the negative consequences of the incident, but now I understand that it has also had positive effects on me. I went to many doctors because of my mental state, but nothing helped. This therapy program has helped me very, very much.” (Participant 11, female).

Some participants expressed this meaning transformation in terms of increased psychological strength and resilience. For example, one participant stated: “I used to think the incident had only a negative impact on me, but today I realize it has made me stronger and more resilient.” (Participant 6, female). Others described a gradual transition from profound hopelessness to cautious acceptance and renewed hope;

“I was hopeless in life; everything felt meaningless. I had forgotten my goals. When I came here, the first sessions were very difficult for me, but after a few sessions, it became easier.” (Participant 7, female).

There was also enhanced appreciation of interpersonal relationships, greater attention to family and friends, improved social connections and tolerance in interpersonal interactions and reduced aggression. For instance, participants noted that; “Since coming here, my behavior with my family and friends has become much better.” (Participant 9, female), “Before, I didn’t think much about friends, but now I understand the value of my friends and family.” (Participant 10, female) and “Before, I was aggressive and always fighting with others, but since attending the therapy sessions, I’ve become much better.” (Participant 4, female). Another participant explained how she now consciously plans time with family members: “Before, when I went to the park with my sister, I would feel tired. Now I’ve decided to spend at least two hours with her on weekends, and if we don’t go to the park, I visit my sister, aunt, or other relatives.” (Participant 9, female). Additionally, a participant noted:

“Since coming to psychotherapy, intimacy at home has increased a lot. I love my parents more, and my behavior has improved. Before, I would quickly argue with children, but now I try to explain things calmly instead of yelling.” (Participant 4, female).

Writing about values, goals, and the future emerged as a key component of this theme. Participants described this process as an opportunity to reassess their life direction and restore a sense of purpose. One participant explained:

“When I failed to get accepted into law school, I became hopeless. But when I came here, I realized there is always a way. I feel much better now and have learned that I need to rise stronger than before. Writing my goals made me feel good, and I have started working toward them.” (Participant 7, female).

Similarly, writing and visualizing future goals were associated with feelings of joy and motivation; “I truly felt good. I had never written or visualized my goals and dreams before, but here I did both, and I felt happy.” (Participant 10, female).

### Theme 4: exposure to traumatic memory and reduction of avoidance

Participants reported that the writing exercises, controlled imagery, and reading personal narratives played a critical role in reducing avoidance of traumatic memories and facilitated gradual exposure to traumatic memories. Participants reported that initial exposure to traumatic memories was difficult and emotionally distressing. However, with repeated practice, emotional intensity decreased and memories became more tolerable; “When I read my story, the incident came back to my mind, but it was not as distressing as before.” (Participant 12, female).

Participants also indicated that gradual exposure reduced fear associated with intrusive mental images and increased emotional tolerance. In some accounts, the opportunity to experience and express previously suppressed emotions—particularly grief—was prominent. One participant noted: “After the incident, I felt a lump in my throat at home and couldn’t cry. But here, during imagery exercises, I cried, and I’m very glad that I was able to cry.” (Participant 13, female). Reductions in behavioral avoidance were also evident. Some participants reported increased willingness to revisit the site of the incident or think about it without severe anxiety; “Now I really want to go to the place of the incident because it gives me a sense of calm, and it feels normal to me.” (Participant 2, female).

### Feasibility

Of the eligible participants, four individuals (all male) discontinued participation after the fifth session due to work commitments or limited availability. Twenty-two participants completed the full intervention. At the three-month follow-up, only 13 participants were available primarily due to Taliban-imposed restrictions on girls’ mobility, migration, or leaving the country for further educational study.

## Discussion

This study aimed to conduct an initial evaluation of the efficacy, acceptability, and processes of change associated with MFT among Afghan youth affected by a terrorist attack. The quantitative findings indicated that MFT was associated with significant reductions in symptoms of PTSD, depression, anxiety, and stress; improvements in cognitive avoidance and cognitive fusion; and increases in posttraumatic growth. Improvements in emotional resilience were modest and should be interpreted as preliminary, given that only the baseline-to-post-intervention comparison reached statistical significance.

One of the distinguishing features of MFT was its adoption of a bottom-up approach to the therapeutic process; that is, the intervention began not with cognitive restructuring, but rather with bodily–emotional regulation. Qualitative findings showed that body-based practices (i.e., deep breathing, relaxation, butterfly hug) served as a primary point of entry into the therapeutic process, providing a sense of safety that enabled gradual access to traumatic memories. This finding is consistent with the trauma literature, which indicates that in complex and war-related trauma, body-oriented interventions are not merely complementary, but rather a prerequisite for cognitive and narrative processing (43).

The significant reduction in PTSD symptoms, along with the relative stabilization of treatment effects, is consistent with memory-based theories of PTSD (13, 15). By integrating graded exposure, narrative writing, autobiographical reconstruction, and bodily regulation, the findings suggest that MFT may have supported trauma memory processing and reduced avoidance, although direct memory integration and the underpinning mechanisms of MFT were not assessed (15). The significant reductions in cognitive avoidance and cognitive fusion represent another key finding, suggesting the importance of cognitive flexibility and acceptance as potential underpinning mechanisms. These results align with the ACT literature and extend previous Afghan findings, which have shown that acceptance-based and cognitive defusion interventions are culturally acceptable and yield meaningful clinical effects (34). Reduced cognitive fusion may have also contributed to decreases in depressive and anxiety symptoms by enabling more flexible and adaptive responses to negative thoughts.

The qualitative findings meaningfully complemented and clarified the quantitative results. One of the most prominent themes was somatic-emotional processing. This finding suggests that in contexts such as Afghanistan—where traumatic experiences are often encoded and stored somatically and sensorily—attention to the body is not merely an adjunctive intervention but a central foundation for psychological coherence. In societies, such as Afghanistan, the expression of trauma and associated emotions is often constrained or suppressed. Participants’ reports of reduced heart palpitations, improved sleep, fewer headaches, and a greater sense of calm following MFT are consistent with the literature on autonomic nervous system regulation and threat system modulation in trauma treatment (43). Notably, caution is warranted when applying tension–release relaxation techniques with war-affected individuals, as the presence of shrapnel or physical injuries may result in additional bodily harm when muscular tension is induced.

Participants also indicated that following MFT they were able to alter their relationship with their thoughts, experiencing thoughts as transient mental events rather than objective realities. This shift—reflected in statements such as “a thought is just a thought”—helps explain the reductions in cognitive fusion and cognitive avoidance observed in the quantitative data. Participants learned to accept thoughts rather than struggle with them and to observe them through mindfulness practices. When combined with memory specificity-focused exercises, these practices contributed substantially to improvements in concentration, memory, and academic functioning, which are consistent with research on increased memory specificity and reduced cognitive anxiety (21, 23, 34, 46, 47, 49).

One of the most distinctive findings of this study was the prominence of growth, meaning, relationships, values, and future orientation. This finding suggests that MFT may have offered benefits beyond symptom reduction and may be associated with the reconstruction of self-continuity across past, present, and future. In a context where Afghan youth face profound educational and occupational foreclosure, focusing on values, future imagery, and the reinterpretation of suffering played a critical role in reducing hopelessness and fostering posttraumatic growth. The significant increase in posttraumatic growth scores in the quantitative data is consistent with these qualitative narratives. Values-based work within ACT, along with future visualization and goal-setting, was described as one of the most engaging components of the intervention. Follow-up interviews conducted with several participants two years after the intervention indicated sustained commitment to personal values and a continued future-oriented perspective, reflecting enduring self-continuity.

Participants also highlighted interpersonal improvements. Reductions in aggression, increased family intimacy, and improved social interactions suggest that the benefits of MFT extended beyond the individual level. This finding complements the METRA and METRA+ (47–49) protocols and highlights the importance of group-based and relational interventions in collectivist cultures, such as Afghanistan. Participants frequently emphasized family and relationships as core values, describing enhanced appreciation for togetherness, mutual support, gratitude, and the opportunity to live meaningfully in the present—features that may be understood as central components of resilience and posttraumatic growth.

Taken together, these findings suggest that MFT may operate through an integrated, sequential pathway in which bodily–emotional regulation, autobiographical memory processing, present-moment acceptance, and future-oriented meaning-making function as interdependent and potentially reinforcing processes. This integrative process—spanning the processing of past trauma, present-moment regulation, and future self-construction—may account for the concurrent reductions in PTSD symptoms and improvements in psychosocial functioning observed in this study. The proposed theoretical model underpinning this pathway, including its conceptual logic and clinical implications, extends beyond the scope of the current paper. A dedicated theoretical and protocol paper elaborating the MFT framework in full is currently in preparation (Ahmadi et al., in preparation). Feasibility findings indicated that although structural, security-related, and economic challenges were present—particularly for working males and for girls under Taliban restrictions—treatment completion rates were relatively acceptable, and participants’ overall experiences of the intervention were predominantly positive. These findings support the potential feasibility of implementing MFT in low-resource and unstable settings. Nonetheless, rigorous future studies are needed both to further evaluate the intervention model itself and to examine the proposed theoretical framework across different populations, trauma types, and cultural contexts.

There are several limitations worth noting. These include the absence of a control group, small sample size, and high attrition at follow-up. Therefore, there is a need for future randomized controlled trials, comparative studies with other trauma-focused interventions, and further investigation of mechanisms of change.

## Conclusion

This study provides preliminary and promising evidence that MFT may offer a potentially effective, integrative, evidence-based, and culturally responsive intervention for Afghan youth affected by trauma. Quantitative findings demonstrated significant reductions in PTSD, depression, anxiety, stress, cognitive avoidance, and cognitive fusion, alongside increased posttraumatic growth. Qualitative findings complemented these results by illustrating participants’ experiences of meaningful changes in bodily–emotional regulation, cognitive processing, meaning-making, future orientation, and interpersonal relationships. Overall, the findings support the notion that simultaneously addressing the past (trauma memory processing), present (emotional regulation and mindfulness), and future (values, meaning, and future imagery) may contribute to the reconstruction of psychological coherence and self-continuity among trauma-exposed youth. By integrating memory-focused, acceptance-based, and body-oriented mechanisms, MFT offers a framework that not only reduces symptoms but also enhances hope, agency, and growth. MFT represents a potentially important step toward developing sustainable, low-intensity, and contextually responsive mental health interventions in war-affected and resource-limited settings.

## Author’s note

The authors sincerely thank Sayed Ali Akbar Sarwary, Sakina Masha, Nasratullah Samim, and all participating Afghan youth, facilitators, Behrawan Research and Psychology Services organization staff, and community partners whose contributions made this project possible.

## Data Availability

The datasets presented in this study can be found in online repositories. The names of the repository/repositories and accession number(s) can be found below: The datasets generated and analyzed during the current study are available in the Harvard Dataverse repository: Ahmadi, S.J. (2026). Replication Data for: Memory-Focused Therapy (MFT) Study. Harvard Dataverse. https://doi.org/10.7910/DVN/MSYTXX.
